# Longitudinal Patterns and Predictors of Cognitive Impairment Classification Stability

**DOI:** 10.1093/arclin/acae107

**Published:** 2024-11-19

**Authors:** Cynthia McDowell, Nicholas Tamburri, Jodie R Gawryluk, Stuart W S MacDonald

**Affiliations:** Department of Psychology, University of Victoria, Cornett Building A236, 3800 Finnerty Road (Ring Rd), Victoria, BC, V8P 5C2, Canada; Institute on Aging and Lifelong Health, University of Victoria, R Hut, Room 103, 3800 Finnerty Road, Victoria, BC, V8P 5C2, Canada; Department of Psychology, University of Victoria, Cornett Building A236, 3800 Finnerty Road (Ring Rd), Victoria, BC, V8P 5C2, Canada; Institute on Aging and Lifelong Health, University of Victoria, R Hut, Room 103, 3800 Finnerty Road, Victoria, BC, V8P 5C2, Canada; Department of Psychology, University of Victoria, Cornett Building A236, 3800 Finnerty Road (Ring Rd), Victoria, BC, V8P 5C2, Canada; Institute on Aging and Lifelong Health, University of Victoria, R Hut, Room 103, 3800 Finnerty Road, Victoria, BC, V8P 5C2, Canada; Division of Medical Sciences, University of Victoria, Medical Sciences Building, Room 104, Victoria, BC, V8P 5C2, Canada; Department of Psychology, University of Victoria, Cornett Building A236, 3800 Finnerty Road (Ring Rd), Victoria, BC, V8P 5C2, Canada; Institute on Aging and Lifelong Health, University of Victoria, R Hut, Room 103, 3800 Finnerty Road, Victoria, BC, V8P 5C2, Canada

**Keywords:** Cognition, Cognitive dysfunction, Dementia, Logistic models, Cognitive reserve, Longitudinal studies

## Abstract

**Objective:**

Classifications such as Cognitive Impairment, No Dementia (CIND) are thought to represent the transitory, pre-clinical phase of dementia. However, increasing research demonstrates that CIND represents a nonlinear, unstable entity that does not always lead to imminent dementia. The present study utilizes a longitudinal repeated measures design to gain a thorough understanding of CIND classification stability patterns and identify predictors of future stability. The objectives were to i) explore patterns of longitudinal stability in cognitive status across multiple assessments and ii) investigate whether select baseline variables could predict 6-year CIND stability patterns.

**Method:**

Participants (N = 259) included older adults (aged 65–90 years) from Project MIND, a six-year longitudinal repeated measures design in which participants were classified as either normal cognition (NC) or CIND at each annual assessment. A latent transition analysis approach was adapted in order to identify and characterize transitions in CIND status across annual assessments. Participants were classified as either Stable NC, Stable CIND, Progressers, Reverters, or Fluctuaters. Multinomial logistic regression was employed to test whether baseline predictors were associated with cognitive status stability patterns.

**Results:**

The sample demonstrated high rates of reversion and fluctuation in CIND status across annual assessments. Additionally, premorbid IQ and CIND severity (i.e., single vs. multi-domain impairment) at baseline were significantly associated with select stability outcomes.

**Conclusions:**

CIND status was unstable for several years following baseline assessment and cognitive reserve may delay or protect against demonstrable cognitive impairment. Further, consideration of cognitive impairment severity at the time of initial classification may improve CIND classifications.

## INTRODUCTION

Despite the growing prevalence rates and associated health care costs, dementia remains the only disease within the top ten causes of death without a steadfast method of prevention or cure (Pike et al., 2022). As dementia is characterized by substantial and irreversible neurological damage, early detection and prevention is paramount. Accordingly, dementia prevention research has spurred an increase in studies aimed at capturing the transitory, pre-clinical phase of dementia. Diagnostic criteria for various cognitive impairment classifications, such as Mild Cognitive Impairment (MCI) and Cognitive Impairment, No Dementia (CIND) have been developed in order to describe this state between normal cognition (NC) and dementia, in which cognitive decline is present beyond what is expected of normal aging but does not yet meet a dementia diagnosis (Petersen, 2004; Welstead et al., 2021). Such classifications are characterized by objective deficits in cognitive abilities that do not impact functional independence (Canevelli et al., 2017; Moradi et al., 2021). Whereas MCI typically requires impaired objective test performance *and* a subjective complaint of cognitive decline, CIND classifications typically require impaired test performance *or* a subjective complaint (Plassman et al., 2011). However, the criteria defining MCI and CIND have become synonymous over time, with each frequently classified using cognitive test performance in addition to a self or informant complaint. It is estimated that ~10–20% of individuals over the age of 65 experience MCI/CIND (Katayama et al., 2020; Makino et al., 2021); however, long-term outcomes following classifications are heterogeneous.

Although originally conceptualized as diagnostic tools to identify those at risk of developing dementia, MCI/CIND represent nonlinear and unstable entities that do not always lead to imminent dementia. In a meta-analysis consisting of 41 studies, Mitchell and Shiri-Feshki (2009) reported an annual conversion rate from MCI to dementia of ~5–10%, with many individuals demonstrating no progression even after a 10-year follow-up. The researchers further reported that the annual conversion rate from MCI specifically to Alzheimer’s disease (ad) is ~7%, and even fewer—approximately 2%—convert to vascular dementia. In contrast, ~37–67% of all MCI cases remain stable MCI without progressing (Pandya et al., 2016). Moreover, a substantial number of individuals with MCI fail to either convert to dementia or even remain MCI at a subsequent time point (Vandermorris et al., 2011); instead, research frequently reveals high rates of *reversion* from MCI back to NC (Aerts et al., 2017; Malek-Ahmadi, 2016; Overton et al., 2019; Pandya et al., 2016; Xue et al., 2019). Thus, while it is true that some individuals classified as MCI/CIND will develop dementia, many will remain consistently MCI/CIND or even revert back to NC.

## VARIABILITY IN REVERSION RATES

A wealth of recent longitudinal studies confirm the common occurrence of reversion from MCI/CIND to NC; however, the estimated prevalence rates of reversion are highly variable. Higher reversion rates are documented in community-based compared to clinic-based cohorts (Manly et al., 2008), as those in clinic-based samples tend to have greater cognitive impairment and increased likelihood of progressing, therefore generating lower reversion rates (Petersen et al., 2014). Previous meta-analyses report reversion rates of ~25–30% in community-based studies, 8–14% in clinic-based studies, and 18–24% when combining the two populations (Canevelli et al., 2016; Malek-Ahmadi, 2016). However, such results are inconsistent in the wider literature. For example, Ganguli et al. (2011) reported that reversion in community-based studies ranged anywhere from ~6–53% over the course of 1 year.

Several other factors may impact reversion rates such that MCI/CIND instability may be due to a variety of subject-based factors (e.g., individual differences in demographics, depression, fatigue at time of testing), diagnostic criteria (e.g., number and type of tests used, cut-off score variations), or study-based factors (e.g., practice effects, measurement error; Malek-Ahmadi, 2016; Moradi et al., 2021; Shimada et al., 2019). Reversion may thus be due to true recovery of illness or neuroplasticity, issues in testing, definition, or individual factors impacting cognitive function such as sleep or nutritional deficits, affect, social anxiety, and more (Canevelli et al., 2016; Moradi et al., 2021). Altogether, conventional classifications such as MCI and CIND—with the intended purpose of predicting dementia progression—are highly susceptible to false positives (Shimada et al., 2019) and may not be indicative of progressive neuropathology. Due to these discrepancies, the prognostic and clinical value of such classifications have been questioned.

## SCOPE FOR IMPROVEMENT

In comparison to the large number of studies that examine predictors of progression, few have focused on such predictors of reversion, and factors related to cognitive impairment stability remain poorly understood (Pandya et al., 2017). Additional studies are needed to understand MCI/CIND stability across longer temporal periods as previous studies have primarily assessed individuals over the course of 1 (e.g., Diniz et al., 2009; Ganguli et al., 2011; Kang et al., 2014; Moradi et al., 2021; Thomas et al., 2019; Tokuchi et al., 2014) or 2 years (e.g., Baiyewu et al., 2002; Sachdev et al., 2013), which is likely not long enough to identify classification stability and whether individuals are truly experiencing persistent, pathological cognitive impairment. In the evolution of dementia, one to two follow-up studies are fallible to misinterpreting both the risk of reversion and conversion, and may obscure the identification of slowly progressing conditions or milder forms of impairment (Aretouli et al., 2013). It is therefore recommended that researchers follow MCI/CIND trajectories for a prolonged temporal period, as the pathological cognitive decline—despite the prolonged prodromal phase of dementia—typically becomes detectable and accelerates after ~4 to 6 years (Hall et al., 2000; Pandya et al., 2017; Wilson et al., 2011).

Additionally, although some studies have indeed employed longer study durations, they remain constrained by the number of follow-up assessments—generally examining individuals only once, or seldom twice, following their baseline assessment. A limited number of studies (e.g., Aerts et al., 2017; Bielak et al., 2010; Overton et al., 2019; Roberts et al., 2014; Sanz-Blasco et al., 2021; Welstead et al., 2021) have evaluated long-term trajectories of MCI/CIND using multiple repeated assessments. Multiple assessments are advantageous to detect the shape of change, distinguish measurement error from true change (Aretouli et al., 2013; Singer & Willett, 2003), and detect fluctuations between classifications. For instance, longitudinal repeated measures studies reveal notable fluctuations in cognitive status over time, whereby a substantial proportion (24–29%) of individuals who reverted to NC later transitioned back to MCI/CIND status at a subsequent assessment (Bielak et al., 2010; Overton et al., 2019; Sugarman et al., 2018). Importantly, individuals who fluctuate between classifications have been previously documented as more prone to dementia progression (Koepsell & Monsell, 2012; Lopez et al., 2012; Roberts et al., 2014). In fact, Petersen (2011) suggests that unstable MCI may represent a pre-condition that will manifest into stable MCI with time. Thus, research remains inconclusive regarding 1) whether individuals classified as stable MCI/CIND stay that way for a long time, and 2) whether individuals who revert to NC remain NC if followed for long enough (Pandya et al., 2016). Ultimately, changes in MCI/CIND status may be missed if 1) stability is characterized based on a single follow-up, 2) too much time has passed without an evaluation, or 3) individuals are not followed for long enough to track long-term outcomes. All told, instability and fluidity between MCI/CIND classifications can jeopardize the utility and validity of the framework, and additional research is required if they are to be deemed sound constructs that can identify individuals at risk of future dementia. Individuals should be followed for several years using multiple repeated assessments to better understand transitions between MCI/CIND and NC, and to accurately identify factors associated with true stability in order to provide a framework for future identification and prevention for at-risk individuals.

## THE CURRENT STUDY

There is a pressing need to enhance the understanding of cognitive impairment classification stability, the non-linear and long-term trajectories, and differences amongst stability predictors, in order to clarify and increase accuracy of the pre-clinical intermittent classification between NC and dementia. Using six-year longitudinal data, the current study sought to gain a better understanding of CIND classification stability across time in order to further its clinical utility as a classification tool. The objectives were to i) explore patterns of longitudinal stability in cognitive status across multiple assessments, and ii) investigate whether select baseline variables could predict 6-year cognitive status stability patterns.

It was anticipated that the overall reversion rates (from CIND to NC) would be higher compared to rates typically reported for clinical populations, but likely lower compared to other community-based reversion studies, as the current study disaggregated those who fluctuated from those who reverted. It was also expected that several patterns of stability (i.e., reversion, stability, progression, fluctuation) would be revealed across the 6 years of study. Such patterns would demonstrate the high levels of instability in CIND classifications and provide preliminary information on the optimal follow-up duration required to identify true CIND stability. Additionally, several baseline predictors were expected to significantly differentiate patterns of stability. In particular, age was hypothesized to significantly predict CIND stability subgroups, such that those who were younger would be more likely to fluctuate or revert in their cognitive status. Further, it was hypothesized that depressive symptoms would be significantly associated with CIND outcomes as depressive symptoms can result in lower cognitive test scores and MCI misclassifications (Ganguli et al., 2009; Overton et al., 2019). However, no specific directional hypotheses were made for depressive symptoms as depression has been associated with stability (Pandya et al., 2017; Sugarman et al., 2018), reversion (Welstead et al., 2021), as well as ad progression (Modrego & Ferrández, 2004). It was also hypothesized that baseline CIND severity (single- vs. multi-domain impairment) would significantly predict subgroup membership. Specifically, those classified as CIND-S (based on impairment in a single cognitive domain) were predicted to have a higher likelihood of reversion and/or fluctuation, whereas those classified as CIND-M (impairment in multiple cognitive domains) were predicted to have a higher likelihood of CIND stability. Such findings would corroborate previous research demonstrating the more labile, unstable nature of single- compared to multi-domain impairment (e.g., Diniz et al., 2009; Loewenstein et al., 2009; Ritchie & Tuokko, 2010; Sachdev et al., 2013). Finally, it was hypothesized that educational attainment and/or premorbid IQ would be negatively associated with CIND stability, providing support for cognitive reserve theory.

## METHODS

### Participants

**Fig. 1:**
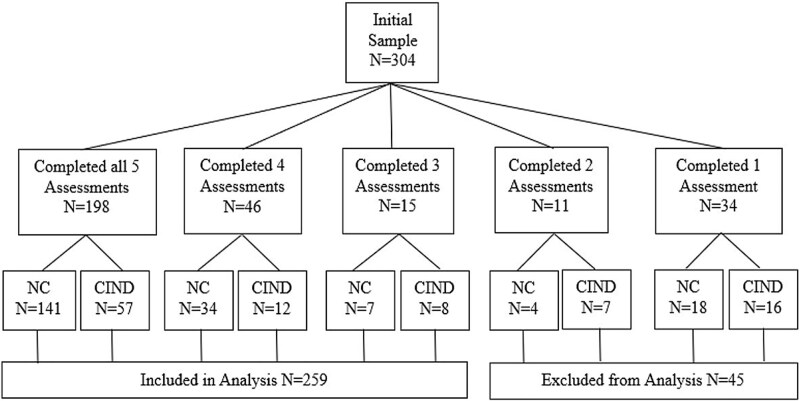
Missing data patterns across the five annual assessments.

The current study examined archival data from Project Mental Inconsistency in Normals and Dementia (MIND), a longitudinal repeated measures study assessing cognitive change in community-dwelling older adults. Project MIND was approved by the University of Victoria Human Research Ethics Board and was conducted in accordance with institutional guidelines. Participants resided in Victoria, bc, and were recruited through local media advertisements soliciting volunteers who were concerned about their cognitive functioning but had not been diagnosed with a neurological disorder. Initial exclusion criteria included physician-diagnosed dementia or an MMSE (Folstein et al., 1975) score less than 24, current psychiatric diagnosis, psychotropic drug use, drug or alcohol abuse, a history of significant head injury (e.g., loss of consciousness), other neurological or major medical illnesses (e.g., Parkinson’s disease, cancer), severe sensory impairment (e.g., difficulty hearing a normal conversation, difficulty reading newspaper-size print), and a lack of English language fluency. The sample initially consisted of 304 participants, however, individuals missing three or more follow-up assessments were excluded from the present study (see Fig. 1). Therefore, data for 259 participants (178 females, 81 males) aged 65 to 90 years (*m* = 73.65; *SD* = 5.71) were included for the present analyses. At baseline assessment, participants were generally well educated (*m* = 15.20; *SD* = 3.08; *range* = 7–24 years of education), performed well on the MMSE (*m* = 28.85; *SD* = 1.11; *range* = 25–30), and were in relatively good health (total number of chronic health conditions: *m* = 2.93; *SD* = 1.93; *range* = 0–10).

### Procedure

Participants were initially screened for inclusion and exclusion criteria via a telephone interview. Baseline testing included a group testing session conducted at the University of Victoria to collect demographic and health data, and an individual testing session conducted in the participant’s home. Within these independent sessions, participants completed various assessments to measure performance across several health domains (e.g., cognitive, psychosocial, physiological). The entire testing battery was repeated annually four times (i.e., Years 1–4), followed by a single follow-up assessment at Year 6 to evaluate change in cognitive status. During each annual testing wave, the measures were identical and the order of presentation did not vary. See MacDonald and Stawski (2020) for a full account of Project MIND, including the incorporation of an intensive repeated measures burst design involving weekly assessments within a given year. Attrition rates for the entire sample (N = 304) were 11.0%, 3.5%, 4.5%, and 9% between Years 1–2, 2–3, 3–4, and 4–6, respectively.

### Measures

#### Cognitive status

CIND status was derived psychometrically each year according to participant’s performance on five cognitive tasks. The cognitive performance tasks consisted of indicators for perceptual speed (WAIS-R Digit Symbol Substitution; Wechsler, 1981), verbal fluency (Controlled Associations; Ekstrom et al., 1976), vocabulary (Extended Range Vocabulary; Ekstrom et al., 1976), episodic memory (Immediate Free Word Recall; Hultsch et al., 1990), and inductive reasoning (Letter Series; Thurstone, 1962). Participants were classified as NC or CIND based upon deficits spanning the five distinct cognitive domains; subjects were classified as CIND if they scored at or below 1.5 SDs relative to age- and education-matched peers on any of the cognitive tasks. Age- and education-based norms were derived from an external sample of 445 adults, aged 65 to 94 years, from the Victoria Longitudinal Study (Dixon & de Frias, 2004). The normative comparison sample was partitioned into four age and education groups (*age* = 65–74 years and 75+ years; *education* = 0–12 years and 13+ years) with means and standard deviations computed for each of the five reference cognitive measures. Given the demographic match, the use of local norms (i.e., Victoria, bc) is preferable for more accurate comparisons across tasks (Bielak et al., 2010). Participants were re-classified each year using an identical and blind classification procedure. Participants classified as CIND at baseline were further subdivided based on CIND severity. Severity was determined according to deficits on a single (CIND-S) cognitive measure vs. multiple (CIND-M) measures (i.e., scoring at or below 1.5 SDs on two or more cognitive measures).

#### Baseline predictors

Predictors of CIND stability outcomes included six variables assessed at baseline: age, sex (0 = *male*, 1 = *female*), CIND severity (CIND-S or CIND-M), depressive symptoms, as well as two indicators of cognitive reserve (premorbid IQ and educational attainment).

##### Depressive symptoms

Symptoms associated with depressive mood were self-reported and assessed using a seven-item questionnaire that asked participants to rate the number of times in the past week they: *could not shake off the blues, felt depressed, felt lonely, felt their life had been a failure, had crying spells, felt fearful, and felt sad*. Items were rated on a four-point scale (0 = *rarely or none of the time (less than 1 day)*, 1 = *some or a little of the time (1–2 days)*, 2 = *occasionally or a moderate amount of time (3–4 days)*, 3 = *most or all of the time (5–7 days)*). A sum score was derived with higher scores indicating greater depressive symptoms.

##### Cognitive reserve

Cognitive reserve was indexed using premorbid IQ as well as total years of educational attainment (*range* = 7–24 years), separately. Estimates of premorbid IQ were computed based on the National Adult Reading Test (NART; Nelson, 1982), as word-reading ability provides a valid indicator of premorbid intellectual functioning for a number of conditions (Bright et al., 2002; Nelson & O'Connell, 1978), and the NART demonstrates high correlation with Full-Scale Intelligence Quotient (FSIQ; Blair & Spreen, 1989). The NART has been standardized for North American populations, demonstrated to accurately predict IQ and brain dysfunction regardless of differences in demographic variables (Bright et al., 2002), and previously utilized as a cognitive reserve predictor of MCI stability (see Osone et al., 2016). The NART is scored out of 61 representing the number of words pronounced incorrectly. These error scores were then transformed into estimated premorbid IQ using the prediction equation (127.8–0.78) produced by Blair and Spreen (1989).

### Statistical procedure

A latent transition analysis (LTA) approach was adapted in order to characterize transitions in CIND status across the annual assessments. Despite latent class analysis typically preceding the use of LTA, the current study utilized the formerly derived CIND status subgroups (i.e., NC or CIND) as the categorical latent variables for each annual assessment. LTA tests the hypothesis of “no change between time-points,” which assumes that status membership at time 2 is the same as status membership at time 1 (Lanza et al., 2010). First, cognitive status membership probabilities were calculated at *t ≥ 1* to reflect the proportion of individuals that belong to either cognitive status group at each time point. Second, transition incidents were examined to identify the timescale in which the sample transitioned from a latent status at *time t* to a different latent status at *time t + 1*. Finally, a matrix of transition probabilities was generated to reflect the percentage of individuals who changed in their cognitive status (NC or CIND) from *t1* to *t2, t3, t4*, and *t6*. Participants were then assigned to one of six outcomes based on their 6-year cognitive status stability pattern:

1) *Stable NC*: Remained NC at each assessment
2) *Progressers*: Progressed from NC and stayed CIND
3) *Stable CIND*: Remained CIND at each assessment
4) *Reverters*: Reverted from CIND and stayed NC
5) *NC Fluctuaters*: Progressed to CIND but returned back to NC at a later assessment
6) *CIND Fluctuaters*: Reverted to NC but returned back to CIND at a later assessment

The second research objective examined baseline predictors of these CIND stability patterns. For the purpose of the present study, in which the intent was to examine (in) stability in CIND, those classified as Stable NC were excluded from these analyses. Additionally, NC and CIND Fluctuaters were collapsed into a single “Fluctuaters” group for majority of the models; however, the model that examined baseline CIND severity utilized the former CIND Fluctuaters subgroup. Multinomial logistic regression with odds ratios was employed to test whether baseline predictors were associated with increased likelihood of being classified as Reverters, Fluctuaters, or Progressers, relative to Stable CIND. Six total models, controlling for age (centered at 74 years) and sex, were fit to investigate the independent predictors, with two-sided significance tests set at *P* < 0.05 for all analyses. Analyses were performed in R Version 4.1.1 (R Core Team, 2021) using various packages, including “nnet” for logistic regressions (Venables & Ripley, 2002).

## RESULTS

### Missing data

The majority of the initial 304 participants completed all five assessments (Years 1, 2, 3, 4, and 6). However, 35% were missing at least one follow-up assessment. A non-significant (*P* > 0.05) Little’s MCAR test suggested that cognitive status data were missing completely at random. Further investigation into the nature of missing values revealed that 53% of the participants who did not return after baseline assessment (i.e., completed one assessment only) were NC, whereas 47% were CIND. Assuming data was missing completely at random, participants missing three or more assessments were excluded from analysis. Therefore, participants who completed at least three assessments—providing the ability to detect fluctuations in cognitive status—were included in the present analyses. See Fig. 1 for a further depiction of missing cognitive status data.

### Stability patterns across years of study

Participants (N = 259) were classified into cognitive status subgroups at each wave of assessment based on their performance on five distinct cognitive domains. Table 1 displays the percentage of individuals classified as NC or CIND for each year of study. At baseline (Year 1), 182 participants were classified as NC and 77 were classified as CIND (see Table 2 for sample characteristics). A chi-square test of independence indicated that baseline cognitive status classifications did not differ by sex, χ^2^(1) = 0.02, *P* = 0.90. Additionally, distinct independent samples t-tests suggested that NC and CIND subgroups did not differ by age or total number of medications (*P >* 0.05); however, those classified as NC demonstrated significantly higher levels of education, *t*(148) = 3.4, *P* < 0.001 as well as scored higher on the MMSE cognitive measure, *t*(123) = 2.2, *P =* 0.03.

**Table 1:** Proportions of cognitive status membership at each assessment

	NC	CIND
Year 1	0.70	0.30
Year 2	0.73	0.27
YEAR 3	0.74	0.26
Year 4	0.75	0.25
YEAR 6	0.76	0.24

**Table 2:** Sample characteristics as a function of baseline cognitive status

	**NC**	**CIND**
Variable	*N = 182*	*N = 77*
Age in years (SD)	73.3 (5.7)	74.5 (5.7)
Sex reported as % females	69	68
Education in years (SD)	15.6 (3.1)	14.2 (3.0)
MMSE score (SD)	29.0 (1.0)	28.6 (1.2)
^a^Medications (SD)	5.8 (3.5)	5.4 (3.3)

Note. NC = Normal Cognition; CIND = Cognitively Impaired, No Dementia. MMSE = Mini Mental State Examination (Folstein et al., 1975).

a Self-reported number of total medications.

**Fig. 2:**
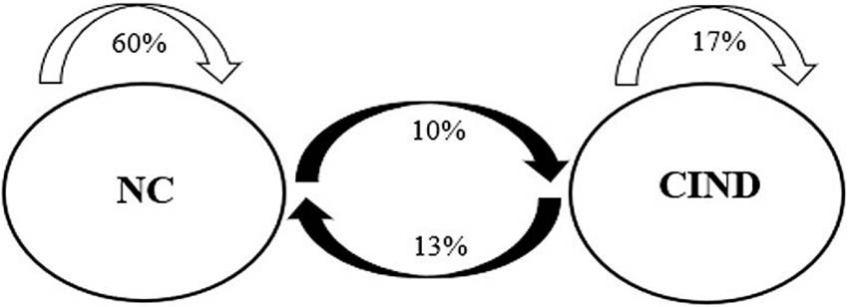
Stability in cognitive status classifications for the sample from years 1 to 2. Note. The white arrows reflect stable classification, the black arrows reflect transitions (i.e., progression or reversion) between cognitive statuses for the entire sample (N = 259).

An LTA framework was then adapted to examine the stability in cognitive status membership across each wave of assessment. Table 3 represents a transition matrix, highlighting the instability in cognitive status. Year 4 demonstrated the greatest discrepancy between baseline classifications and follow-up assessment; the majority (51%) of those classified as CIND at baseline, as well as 16% of those classified as NC, changed cognitive status’ by Year 4. A further investigation revealed that the majority (29%) of all transitions in the sample occurred at Year 2, followed by 28% of all transitions taking place at Year 3, 25% at Year 4, and 18% at Year 6. Figure 2 represents the total sample’s stability and transition patterns between Years 1 to 2.

**Table 3:** Transition matrix displaying cognitive status parameters compared to baseline

Baseline		Follow-up	
		Status 1: NC	Status 2: CIND
STATUS 1: NC *(N = 182)*	Year 2	**0.85**	0.15
	Year 3	**0.86**	0.14
	Year 4	**0.84**	0.16
	Year 6	**0.88**	0.12
status 2: CIND *(N = 77)*	Year 2	0.43	**0.57**
	Year 3	0.43	**0.57**
	Year 4	0.51	**0.49**
	Year 6	0.44	**0.56**

Of the initial 182 participants classified as NC at baseline, 126 (69%) of these individuals remained NC even as long as 6 years following baseline assessment. However, 56 (31%) progressed to CIND and of these, 19 (34%) remained CIND while 37 (66%) reverted back to NC at a later assessment. Comparatively, of the 77 participants classified as CIND at baseline, 25 (32%) remained stable CIND across each annual assessment, whereas 52 (68%) reverted back to NC. However, more than half (56%) of these participants who reverted back to NC returned to CIND at a subsequent assessment. Participants were classified into one of six subgroups to reflect these cognitive status stability patterns: i) Stable NC (N = 126), ii) Progressers (N = 19), iii) Stable CIND (N = 25), iv) Reverters (N = 23), v) NC Fluctuaters (N = 37; classified as NC at baseline), and vi) CIND Fluctuaters (N = 29; classified as CIND at baseline). Table 4 presents descriptive statistics at baseline for each stability group’s performance across the five neuropsychological tasks as well as additional predictor variables.

**Table 4:** Baseline Descriptive Statistics by Stability Group Across Neuropsychological Tasks and Predictor Variables

*Mean Raw Scores (SDs) at Baseline*	**Stable NC** *N = 126*	**Stable CIND** *N = 25*	**Progressers** *N = 19*	**Reverters** *N = 23*	**NC Fluctuaters** *N = 37*	**CIND Fluctuaters** *N = 29*
Verbal Fluency	15.8 (4.9)	9.4 (3.6)	14.4 (5.8)	13.1 (4.5)	13.5 (4.9)	11.3 (4.3)
Episodic Memory	18.3 (3.7)	13.7 (4.0)	15.1 (2.9)	16.7 (4.1)	16.3 (3.1)	14.5 (4.7)
Inductive Reasoning	11.8 (3.3)	3.8 (4.6)	8.1 (3.8)	7.3 (5.1)	10.1 (3.4)	5.3 (4.4)
Perceptual Speed	45.9 (8.8)	34.1 (11.9)	39.0 (9.4)	34.65 (11.9)	43.4 (10.0)	40.4 (7.5)
Vocabulary	58.9 (5.1)	49.3 (9.3)	57.0 (5.7)	55.8 (7.9)	55.5 (8.0)	51.8 (8.9)
Age (years)	73.1 (5.1)	75.9 (6.1)	76.3 (7.3)	72.4 (4.3)	72.5 (5.7)	75.0 (6.1)
Depressive Symptoms	8.3 (2.6)	10.1 (4.7)	9.1 (3.5)	8.0 (2.9)	8.7 (2.7)	9.3 (3.1)
Premorbid IQ	119.0 (4.6)	109.4 (9.7)	118.8 (5.1)	113.8 (7.5)	116.4 (5.9)	113.7 (7.6)
Education (years)	15.2 (3.2)	14.1 (1.8)	15.7 (3.0)	14.2 (3.8)	15.3 (2.6)	14.4 (3.0)
MMSE	29.0 (1.0)	28.6 (1.3)	28.8 (1.1)	28.8 (1.3)	28.7 (1.1)	28.4 (1.2)

Verbal Fluency (total correct synonyms generated in 6 min) assessed using the Controlled Association task (Ekstrom et al., 1976); Episodic Memory (total correct /30) assessed using the Immediate Free Word Recall task (Hultsch et al., 1990); Inductive Reasoning (total correct /20) assessed using the Letter Series task (Thurstone, 1962); Perceptual Speed (total correct responses in 90s) assessed using the WAIS-R Digit Symbol Substitution task (Wechsler, 1981); Vocabulary (total correct definitions generated in 10 min) assessed using the Extended Range Vocabulary task (Ekstrom et al., 1976); Depressive symptoms assessed using sum scores for a seven-item questionnaire indexing depressive mood in the past week, with individual items rated on a four-point scale ranging from 0 (rarely or none of the time (less than 1 day)) to 3 (most or all of the time (5–7 days)); Error scores on the National Adult Reading Test (NART) were transformed into estimated premorbid IQ using the prediction equation (127.8–0.78; Blair & Spreen, 1989); Global cognitive functioning assessed using the Mini Mental State Examination (MMSE; Folstein et al., 1975).

### Baseline predictors of 6-year stability outcomes

The second research objective examined whether select baseline variables could independently predict 6-year cognitive status stability. For the purpose of the present study, those classified as Stable NC were not included in these analyses. Additionally, NC and CIND Fluctuaters were collapsed into a single “Fluctuaters” group (N = 66) such that regression models compared Progressers, Reverters, and Fluctuaters to Stable CIND. Of note, for the CIND severity predictor, the sample was restricted to those classified as CIND at baseline (N = 77). The variables included age, sex, depressive symptoms, CIND severity (N = 47 CIND-S; N = 30 CIND-M), years of education, and premorbid IQ. Table 5 contains the parameter estimates for each model. Baseline premorbid IQ and CIND severity significantly distinguished between select CIND stability subgroups. Those with higher premorbid IQ scores at baseline had a significant 10% (*P <* 0.01) and 21% (*P* < 0.001) increase in the odds of being classified as Fluctuaters and Progressers, respectively, compared to those classified as Stable CIND. Additionally, of those classified as CIND at baseline, Fluctuaters and Reverters were more likely to be classified as CIND-S, whereas the Stable CIND subgroup was more likely to be classified as CIND-M. That is, individuals with higher CIND severity at baseline were significantly less likely to fluctuate (*P =* 0.01) or revert (*P* = 0.01) in their CIND status. Rather, 72.4% and 73.9% of participants classified as CIND Fluctuaters and Reverters, respectively, were classified as CIND-S at baseline. Further investigation into overall individual model fit revealed that both the premorbid IQ [χ^2^(12) = 26.08, *P* < 0.01; McFadden *R*^2^ = 0.08] and CIND severity [χ^2^(8) = 15.60, *P <* 0.05; McFadden *R*^2^ = 0.09] models (each controlling for age and sex) were predictive of overall stability classifications. The premorbid IQ model correctly classified 52.6% of participants, with 10.0% of Progressers, 0% of Reverters, 92.4% of Fluctuaters, and 28.0% of Stable CIND being correctly classified. Similarly, the CIND severity model correctly classified 52.0% of participants, with 34.8% of Reverters, 58.6% of Fluctuaters, and 60.0% of Stable CIND being correctly classified.

**Table 5:** Logistic regression models with stable CIND as referent group

	Reverters (N = 23)			Fluctuaters (N = 66)					Progressers (N = 19)			
	b	95% CI	SE	Exp(b)	b	95% CI	SE	Exp(b)	b	95% CI	SE	Exp(b)
Age	−0.10	−0.20, 0.00	0.05	0.90	−0.06	−0.14, 0.01	0.04	0.94	0.01	−0.08, 0.10	0.05	1.01
Sex	−0.46	−1.65, 0.74	0.61	0.63	0.03	−0.97, 1.03	0.51	1.03	−0.22	−1.47, 1.04	0.64	0.80
CIND severity	−1.68^*^	−3.01, −0.35	0.68	0.19	−1.52^*^	−2.72, −0.32	0.61	0.22	—	—	—	—
Depression	−0.18	−0.40, 0.03	0.11	0.84	−0.07	−0.20, 0.05	0.06	0.93	−0.07	−0.25, 0.11	0.09	0.93
*Cognitive Reserve*												
Premorbid IQ	0.06	−0.02, 0.13	0.04	1.06	0.09^**^	0.03, 0.16	0.03	1.10	0.19^**^	0.08, 0.29	0.05	1.21
Education	−0.05	−0.26, 0.15	0.10	0.95	0.07	−0.10, 0.24	0.09	1.07	0.21	−0.01, 0.44	0.12	1.23

*Note.* CIND=Cognitively Impaired, Not Demented; CI=Confidence Interval; Depression = Symptoms Consistent with Depressive Mood; Stable CIND was coded as 0, Reverters coded as 1, Fluctuaters coded as 2, and Progressers coded as 3.

For CIND severity, Fluctuaters included only those classified as CIND at baseline (N = 29).

All models controlled for both Age and Sex.

^**^ *P <* 0.001;

^*^ *P* < 0.05.

## DISCUSSION

The present study was designed to add to the existing cognitive impairment classification literature by utilizing a 6-year study with multiple repeated assessments to gain a more thorough understanding of longitudinal CIND classification stability patterns, and to identify predictors of future stability. The primary findings show high rates of both reversion and fluctuation in CIND status. Additionally, logistic regression analyses with odds ratios identified baseline CIND severity and premorbid IQ as predictors of future CIND stability patterns.

### Patterns of CIND stability across years

In this community-based sample, 68% of those classified as CIND at Year 1 reverted from CIND back to NC at some point across the 6-year study. However, the majority of these individuals eventually transitioned back to CIND at a later assessment—highlighting the evident occurrence of CIND status fluctuation. In fact, the most common long-term stability pattern for those classified as CIND at baseline was indeed fluctuation, with 38% fluctuating between CIND and NC. Similar results were reported by Sugarman et al. (2018) in which 29% of individuals who reverted eventually transitioned back to MCI at a follow-up assessment. Such findings suggest that those who improve in their cognitive status remain at risk of re-transitioning to CIND if followed for long enough. Furthermore, fluctuation was apparent not only for those classified as CIND at baseline, but also for those classified as NC, generating a 26% fluctuation rate for the entire sample of older adults. This is an important finding as few studies have followed individuals for long enough to account for such fluctuations, and instability/fluidity in the literature is likely underestimated. Correspondingly, the current study’s true reversion rate (i.e., reverted to NC and stayed NC) was 30%. This is consistent with previous meta-analyses that report reversion rates of ~25–30% in community-based studies (Canevelli et al., 2016; Malek-Ahmadi, 2016). However, this was higher than predicted; it was expected that the rate of reversion would be lower given that the current study separately considered and extracted those who fluctuated from those who reverted. Further, Project MIND is composed of a relatively healthy sample, and it was anticipated that initial CIND classification would reflect stable pathological cognitive impairment rather than momentary poor cognitive performance due to external factors such as socioeconomic status. For instance, Welstead et al. (2021) followed a comparatively healthy sample for 6 years and reported a 7% reversion rate. Nonetheless, the current high reversion rate suggests that CIND may not represent the prodromal stage of dementia and provides further justification for continued research on factors associated with CIND reversion versus stability.

Previous studies have predominately examined cognitive impairment stability by employing a 1-year study period and/or a single follow-up assessment that is likely to produce more inaccurate stability patterns. The current study demonstrated that most transitions between cognitive statuses occurred at Years 2 and 3 (i.e., 1- and 2-years following baseline assessment). Due to these transitions, cognitive status classifications by Year 4 were the least comparable to Year 1 classifications, suggesting that CIND status is likely to be unstable for at least 4 years following baseline assessment. In fact, 51% of those classified as CIND at baseline were no longer classified as CIND at Year 4. Such findings may explain the large range of reversion rates reported in the literature, as transitions from CIND to NC occur at disparate points in time. The present findings also corroborate previous research demonstrating that a substantial portion of individuals classified as CIND (and synonymous classifications) will fluctuate in their cognitive status, reverting back to NC at subsequent assessments. Such variability in CIND classifications may disrupt the development of effective interventions for individuals with dementia, as inclusion of those who are on a reverting trajectory in research and clinical trials can bias findings (de Jager & Budge, 2005; Sugarman et al., 2018). Predicting stable CIND—representing an at-risk subgroup—across time may thus improve clinical trial research and the interpretation of results. Given the findings of the current study, and consistent with previous recommendations (see Pandya et al., 2017), it is suggested that researchers and clinicians assess individuals annually for at least 4 years in order to better determine and detect CIND stability.

### Predictors of CIND stability outcomes

Neither age nor sex (as independent predictors) were significantly associated with increased likelihood of reverting, progressing, or fluctuating relative to CIND stability. This is in contrast to expectations in which age specifically was predicted to be associated with stability outcomes; previous studies have demonstrated that younger ages are more likely to recover from MCI back to NC (Gao et al., 2014; Xue et al., 2019), the prevalence of CIND classifications increase with age (Plassman et al., 2011), and older age is associated with increased incidence of progression from MCI to ad (Xue et al., 2019). However, the current findings are consistent with other research also reporting no significant differences between MCI stability and reversion for age (Hu et al., 2020; Sachdev et al., 2013) or sex (Gao et al., 2014; Hu et al., 2020). Likewise, no significant relationships were observed between CIND stability outcomes and depressive symptoms.

#### Single vs. multiple domain impairment for classification accuracy

Baseline CIND severity (i.e., CIND classified based on single vs. multiple domains) was associated with a decreased odds of being classified as either Reverters or Fluctuaters, such that those with greater severity (i.e., CIND-M) were more likely to be classified as CIND across all 6 years of evaluation. This corresponds with several previous studies indicating that individuals are more unlikely to revert to NC with multi-domain cognitive impairment compared to single domain (Han et al., 2012; Loewenstein et al., 2009; Makino et al., 2021; Manly et al., 2008). Importantly, compared to subjects with less impairment, studies have shown that those with multi-domain cognitive impairment are also more likely to progress to dementia (Aretouli et al., 2013; Manly et al., 2008). In their study, Ritchie and Tuokko (2010) reported that, while other CIND classifications (e.g., amnestic vs. non-amnestic CIND) demonstrated poor sensitivity for dementia conversion, CIND-M was the only diagnostic criteria to significantly predict dementia 5 years later. The current study provides additional evidence that future stability in CIND status may be better predicted when the initial classification is derived from assessment of multiple cognitive domains (Han et al., 2012; Ritchie & Tuokko, 2010).

It has been suggested that individuals who are more variable in their cognitive course may be at higher risk of dementia progression, such that CIND fluctuation may represent an underlying pathological precondition (Lopez et al., 2012; Pandya et al., 2016; Petersen, 2011; Roberts et al., 2014). However, a final follow-up investigation revealed that the majority (72.4%) of those classified as CIND Fluctuaters were classified as CIND-S at baseline. Likewise, 73.9% of those classified as Reverters were also CIND-S at baseline. Therefore, it is likely that this fluctuation group was not a result of an underlying mechanism that will lead to eventual pathological cognitive decline, but rather a result of the transient, labile nature of CIND-S classifications (e.g., due to factors such as lifelong low performance). Classifying cognitive impairment based on poor performance on a single cognitive task may misclassify low-performing but otherwise cognitively intact participants (Brodaty et al., 2013). While CIND-S is subject to fluctuations and reversions, producing diagnostic (and prognostic) ambiguity, CIND-M classifications may be indicative of “at-risk” individuals experiencing true pathological decline. In effect, CIND classifications could be maximized by considering CIND severity at initial assessment such that multi-domain impairment should be warranted in the initial classification criteria.

We further recommend that future research investigate whether specific neuropsychological domains—such as processing speed, vocabulary, and episodic memory (including immediate vs. delayed recall measures) —contribute unique predictive value for CIND stability and dementia progression, especially within the framework of CIND-M classifications. A comprehensive understanding of the domains that most reliably indicate stable impairment will enhance the precision of prognostic assessments for individuals vulnerable to cognitive pathology. Moreover, adopting a “comprehensive criteria” approach (see Jak et al., 2009), which requires multiple measures of impairment *within* a given cognitive domain, may further refine stability classifications and increase the clinical relevance of CIND assessments. Notably, this discrepancy—where academic researchers often classify cross-domain multi-impairment whereas clinicians and clinical researchers emphasize within-domain multi-impairment—may be a key factor contributing to the significant variations in stability and reversion rates reported in the literature. That is, classifying and examining multiple indicators of impairment within cognitive domains may potentially enhance the consistency of stability and reversion findings across studies but also improve the clinical utility of CIND classifications, making them more reliable and applicable in both research and practice.

#### Cognitive reserve metrics for predicting stability

Higher educational attainment has been previously associated with recovery from MCI to NC and it has been proposed that individuals with higher education levels may better understand and implement health knowledge and lead healthier lifestyles, thereby reducing MCI risk (Pandya et al., 2017; Xue et al., 2019). However, educational attainment within the current study—although significantly differentiating baseline cognitive status—was not associated with stability outcomes. In contrast, premorbid IQ was significantly associated with increased likelihood of being classified as Fluctuaters and Progressers relative to Stable CIND. This is consistent with Osone et al. (2016) who similarly found that, while years of education did not significantly differentiate MCI subgroups, premorbid IQ was significantly higher in those who reverted. It was suggested that, compared to premorbid IQ—a more sensitive measure of cognitive reserve—educational attainment may have limited variance and is thus less sensitive to detecting an effect. The results suggest that higher baseline premorbid IQ may delay or protect against recurrent CIND classification due to cognitive reserve. Individuals with greater reserve are posited to have a greater defense against the cognitive decline process prior to reaching a diagnosis threshold (Sachdev et al., 2013). For instance, in the current study, individuals were more likely to delay their CIND classification (i.e., be classified as NC at baseline) and progress at a later assessment compared to being classified as CIND at Year 1 (i.e., Stable CIND), independent of age or sex differences. Overall, higher baseline premorbid IQ, denoting greater cognitive reserve, may have exhibited protective effects against stable cognitive impairment. However, few studies have examined premorbid IQ and further research should explore this variable as an important predictor of long-term CIND stability.

### Clinical and practical considerations

There is a pressing need for ongoing dementia prevention studies and methods for predicting those at risk of pathological decline. While dementia detection may be facilitated by the identification and quantification of select biomarkers (e.g., B-amyloid, tau-proteins) and neuroimaging techniques, these can be costly and invasive. Alternatively, routine screening for CIND status may represent a non-invasive and inexpensive tool for dementia detection. The identification of an at-risk group may contribute to early detection strategies for slowing cognitive decline. Early intervention of dementia—before prominent neuronal damage and notable symptomatology occurs—is generally viewed as most effective (Pike et al., 2022), and impairment in multiple cognitive domains may represent an early and reliable marker for pathological impairment. The identification of individuals more likely to show positive outcomes (i.e., reversion) can also benefit the healthcare system by better allocating health resources; CIND stability information may inform decisions about the frequency of future contact (e.g., decrease transitory CIND-related visits) and the necessary monitoring required. Focused attention can thus be given to those who exhibit adverse trajectories (i.e., CIND stability) which may decrease burden for individuals, care-partners, and the larger healthcare system (Plassman et al., 2011).

Additionally, a false positive CIND classification, while not a formal diagnosis, can cause considerable anxiety and feelings of uncertainty. Misclassifications may also lead to additional unintended negative consequences including stigmatization, discrimination (Canevelli et al., 2017), and can alter people’s self-perceptions as well as life decisions (e.g., moving, retiring, looking into care facilities; Thomas et al., 2019). Moreover, if CIND is viewed as an imminent, unavoidable dementia indicator, then it may trigger the start of targeted yet unnecessary and potentially harmful treatment, particularly if pharmaceuticals are involved (Sugarman et al., 2018). However, rather than viewing CIND as an irreversible state leading to imminent dementia, the current study lends further support to the supposition that CIND—in particular, CIND-S—is a reversible, labile state. Illuminating the inherent instability in CIND classifications, even as long as 6 years following baseline assessment, demonstrates the heterogeneity in long-term outcomes and may help reduce over-diagnosis, stigma, anxiety, and over-medicalization. Further, identifying factors associated with future CIND stability may allow clinicians to better predict those at risk of dementia, as well as better inform their patients by providing a realistic prognosis and treatment recommendations.

### Strengths and limitations

The current study has several notable strengths including the study duration and number of repeated assessments; the four follow-up assessments (in addition to the initial baseline assessment) and the extensive 6-year study duration affords a more comprehensive investigation and understanding of CIND stability. Second, the current sample is composed of a relatively homogenous population of healthy individuals residing in the same geographical area. Thus, participants likely have similar life experiences such as education and socioeconomic status; using an alternatively heterogeneous sample to investigate CIND status stability would likely impact the accuracy of results and make predictions less precise (Pandya et al., 2017). Therefore, the sample homogeneity is ideal for modeling aging characteristics, as a broader and more diverse sample may complicate analyses due to cohort effects (Welstead et al., 2021). Third, the use of a community-based sample is advantageous to avoid the inherent cognitively impaired selection bias of clinic-based samples and is more representative of the general public (Ganguli et al., 2011; Ritchie & Tuokko, 2010). It has also been suggested that validating research criteria at the community level may be beneficial prior to incorporating into clinical practice (Ganguli et al., 2011). Fourth, whereas the majority of studies within this literature have examined the recovery *rate* of MCI to NC but not recovery *factors* (Xue et al., 2019), the present study examined predictive factors for reversion as well as progression and fluctuation, relative to stability. Additionally, the current study is amongst the few that has examined the protective role of cognitive reserve in the context of MCI/CIND stability, and amongst the few specifically examining CIND stability. That is, the limited existing research on instability predominately leverages MCI classifications, despite the potential value of exploring other classifications; given the heterogeneous reversion rates reported in the literature, it is important to study different definitions to help increase overall clinical utility.

However, the current study is not without limitations. First, despite the strengths of the healthy and homogenous sample, this may also restrict generalizability to the larger population. Nevertheless, although the sample was Caucasian, a previous CIND study reported no significant association between race and the incidence of CIND or dementia (Plassman et al., 2011). A second limitation is the small cell sizes of CIND outcomes. Despite the reasonably large initial sample size, restricting analyses to those with three or more assessments and subsequently dividing the sample into different CIND stability outcomes resulted in smaller cell sizes that may have limited power to detect effects. Third, the current study was not able to determine which individuals eventually transitioned from CIND to dementia. Thus, although it is speculated that those who remained stable CIND represent an “at risk” group, it was ultimately not possible to confirm this. Additionally, while symptoms consistent with depressive mood were utilized to delineate depressive symptoms, this operationalization may inherently limit the depth of our understanding. Future investigations should hence examine the predictive role of depression on CIND stability by employing more established and validated depression assessment tools. Finally, the CIND outcomes were not fully validated as participants were not formally evaluated by a clinician. Whereas the neuropsychological measures were selected to broadly capture a continuum of cognitive functioning in a community-based sample, the absence of additional domain-specific tasks (such as semantic fluency, delayed recall, and visuospatial measures) may limit the sensitivity and comprehensiveness of clinical CIND classifications. Although subjects were recruited based on an expressed subjective concern about their cognitive functioning (a common criteria used in clinical assessments of CIND), and the use of objective cognitive tests has shown to be relatively accurate and robust (Malek-Ahmadi, 2016), future research should include and investigate the impact of domain-specific cognitive tasks to enhance the detection and characterization of cognitive impairment.

## CONCLUSION

Cognitive impairment classifications such as CIND are characterized by prognostic and clinical heterogeneity. The current study exemplifies the considerable variability that exists across long-term outcomes, with several individuals reverting or fluctuating in their cognitive status across time. However, the relatively small number of individuals who remained stable CIND over the course of the study may provide important evidence for the identification of risk and protective factors, as well as implications for future research and targeted clinical management. The primary findings suggest that most individuals are unstable in their cognitive status for several years following baseline assessment, and factors such as cognitive reserve may delay or protect against detectable cognitive impairment. Moreover, considering cognitive impairment severity (i.e., single- vs. multi-domain impairment) at the time of initial classification may improve CIND classifications. Due to the large number of individuals living with CIND or dementia, and the projected increase in these numbers, even modest improvements in early detection or prevention procedures can have long and lasting impacts on the healthcare system and quality of life.
